# A new species of *Euscorpius* Thorell, 1876 (Scorpiones, Euscorpiidae) from Marmara Region of Turkey

**DOI:** 10.3897/zookeys.281.4732

**Published:** 2013-03-28

**Authors:** Ersen Aydın Yağmur, Gioele Tropea

**Affiliations:** 1 Alaşehir Vocational School, Celal Bayar University, Manisa, Turkey; 2 Società Romana di Scienze Naturali, Rome, Italy

**Keywords:** Scorpion, *Euscorpius*, new species, Turkey

## Abstract

A new species of the genus *Euscorpius* Thorell, 1876 is described based on specimens collected from Bursa Province, in Marmara Region of Turkey. It is characterized by a mesotrichous trichobothrial pattern (*Pv*= 8, *et*= 6, *em*=4, *eb*= 4), medium size and light coloration. *Euscorpius (Euscorpius) rahsenae* **sp. n.** is the second species of the subgenus *Euscorpius* recognizedin Turkey.

## Introduction

The genus *Euscorpius* Thorell, 1876 is one of the most studied taxa of scorpions, however, because of its complexity, its taxonomy continuously changes and is not completely clear, especially in the Balkans, Turkey and in Western Europe. The *Euscorpius* populations of Turkey have been poorly studied, and only three valid species are recognized: *Euscorpius (Polytrichobothrius) italicus* (Herbst, 1800), *Euscorpius (Alpiscorpius) mingrelicus* (Kessler, 1874) and *Euscorpius (Euscorpius) avcii* Tropea et al. 2012. *Euscorpius mingrelicus*, which is a species complex has six described subspecies in Turkey [*Euscorpius mingrelicus mingrelicus* (Kessler, 1874), *Euscorpius mingrelicus ciliciensis* Birula, 1898, *Euscorpius mingrelicus phrygius* Bonacina, 1980, *Euscorpius mingrelicus ollivieri* Lacroix, 1995, *Euscorpius mingrelicus legrandi* Lacroix, 1995, and *Euscorpius mingrelicus uludagensis* Lacroix, 1995)] that need clarification.

Presence of the subgenus *Euscorpius* in Turkey have been reported many times, under the name of *Euscorpius carpathicus* or *Euscorpius carpathicus* “complex” from İstanbul (Hadži 1930); Havza (Samsun) (Schenkel 1947); Acıpayam and Honaz Mountain (Denizli), Eğridir (Isparta), Korikos (Mersin) and İstanbul (Vachon 1951); Sinop (Tolunay 1959); Amasya, the Middle Taurus, Borçka (Artvin), Çanakkale, Trakya and Efes (İzmir) (Kinzelbach 1975, 1982); Alanya (Antalya), Bursa Town and Gemlik (Bursa), Ayvacık and Çan (Çanakkale), Sarıyer, Üsküdar and Büyükada Island (İstanbul), Urla (İzmir), Fethiye (Muğla), Sinop Town and Ada vicinity (Sinop) (Karataş 2006); and Dilek Peninsula (Aydın) (Koç and Yağmur 2007). Furthermore, Kinzelbach (1975) recorded *Euscorpius mesotrichus* from Şile (İstanbul) and Prinkipos Island (Büyükada Island) in the Marmara Sea. Further studies (Di Caporiacco 1950; Fet 1997; Fet and Braunwalder 2000; Gantenbein et al. 2001; Fet and Soleglad 2002; Fet et al. 2003; Tropea et al. 2012; Tropea and Rossi 2011-2012) reported that *Euscorpius mesotrichus* is not an available name, and populationswithin Kinzelbach’s interpretation, referred to other species such as *Euscorpius tergestinus*, *Euscorpius balearicus*, *Euscorpius sicanus* and other forms. Recently, Tropea et al. (2012) described *Euscorpius avcii*, the first valid species of the subgenus *Euscorpius* in Turkey from Dilek Peninsula.

The new species described herein, *Euscorpius (Euscorpius) rahsenae* sp. n. is the second species of the subgenus *Euscorpius* s.str. in Turkey.

## Materials and methods

A total of 59 specimens belonging to the new species were collected from Bursa Province, in the Marmara region of Turkey. Comparison material: *Euscorpius avcii*, holotype ♂, Dilek Peninsula National Park, Canyon, Dilek Peninsula, near Davutlar Town, Kuşadası, Aydın, Turkey, 07.10.2005, leg. H. Koç (MTAS); paratypes, 1 ♂, 5 ♀♀, Dilek Peninsula National Park, Canyon, Dilek Peninsula, near Davutlar Town, Kuşadası District, Aydın Province, Turkey, 07.10.2005, leg. H. Koç (MZUF); same data, 1 ♂, 2 ♀♀ (GTC); Abbreviations: *V*: trichobothria on ventral pedipalp chela manus; *Pv*: trichobothria on patella ventral surface; *Pe*: trichobothria on the pedipalp patella external surface; *et*: external terminal; *est*: external subterminal; *em*: external medium; *esb*: external suprabasal; *eba*: external basal *a*; *eb*: external basal; DPS: dorsal patellar spur; DD: distal denticle; MD: median denticles; OD: outer denticles; ID: inner denticles; IAD: inner accessory denticles; MZUF: Museo Zoologico ‘La Specola’ dell’Università di Firenze, Florence, Italy; GTC: private collection of Gioele Tropea, Rome, Italy; MTAS: Museum of the Turkish Arachnological Society; MSNB: Museo Civico di Scienze Naturali “E. Caffi”, Bergamo, Italy; ZMSU: Zoology Museum of Sinop University, Turkey; KUAM: Arachnological Museum of Kırıkkale University, Turkey; AZM: Alaşehir Zoological Museum, Celal Bayar University, Manisa, Turkey; FKCP: František Kovařík Collection, Praha, Czech Republic.

The trichobothrial notations follow Vachon (1974). The morphological measurements are given in millimeters (mm) following Stahnke (1970). The morphological nomenclature follows Stahnke (1970), Hjelle (1990) and Sissom (1990); the chela carinae and denticle configuration follows Soleglad and Sissom (2001) and sternum terminology follows Soleglad and Fet (2003); description and terminology of hemispermatophore follows Soleglad and Sissom (2001) and Fet and Soleglad (2002).

## Taxonomy

### Family Euscorpiidae Laurie, 1896. Genus *Euscorpius* Thorell, 1876. Subgenus *Euscorpius* Thorell, 1876

#### Euscorpius rahsenae

Yağmur & Tropea sp. n.

urn:lsid:zoobank.org:act:9D78689F-F701-4CD5-9B79-A58BA80D88A4

http://species-id.net/wiki/Euscorpius_rahsenae

##### Type material.

**Holotype:** 1♂, Tirilye Village, Mudanya District, Bursa Province, Turkey, 06.07.2012, 40°23'08.9"N, 28°48'20.9"E, 39 m, Red Pine Forest, leg. R.S. Kaya & H. Koru (AZM).

**Paratypes: 1.** 1♀. Beşevler Neighborhood, Nilüfer District, Bursa Province, 23.06.2004, 21.04.2012, 40°12'46"N, 28°57'58"E, 140 m, leg. R.S. Kaya (AZM). **2.** 1♀. Beşevler Neighborhood, Nilüfer District, Bursa Province, 05.05.2005, 40°11'47"N, 28°57'58"E, 153 m, leg. R.S. Kaya (AZM). **3.** 3♀♀. Yalıçiftlik Village, Ruined Building, Mudanya District, Bursa Province, 21.04.2012, 40°21'16"N, 28°42'58"E, 97 m, leg. H. Koru (AZM). Same data,1♂, 23.10.2012. **4.** 1♂, 1♀. Tirilye Village, Mudanya District, Bursa Province, 17.06.2012, 40°23'08.9"N, 28°48'20.9"E, 39 m, leg. E.A. Yağmur & R.S. Kaya (GTC). Same data, 6♀♀ (AZM). Same locality 4♂♂, 3♀♀, 06.07.2012, leg. R.S. Kaya & H. Koru; 3♂♂, 7♀♀, 22.09.2012, leg. R.S. Kaya & H. Koru (GTC). Same data 1♂, 1♀ (MSNB). Same data 2♂♂, 9♀♀ (AZM). Same locality 2♂♂, 8♀♀, 06.11.2012, leg. R.S. Kaya & H. Koru (AZM), 1♂, 1♀ (FKCP). **5.** 1♂, 1♀. Çiftehavuzlar Neighborhood, Karadeniz Street, Osmangazi District, Bursa Province, 28.10.2012, 40°12'30"N, 29°03'05"E, 110 m, Home garden, leg. H. Koru (AZM).

##### Etymology.

The specific epithet refers to Dr. Rahşen S. Kaya, a Turkish arachnologist, for her friendship and kind contributions to collecting scorpions.

##### Diagnosis.

A medium *Euscorpius* species, total length 27–34 mm.Color of adults very light brown-yellowish with carapace and pedipalpslittle darker, legs, telson and chelicerae lighter. Carinae dark, distinctly brownish-blackish, especially on pedipalps. Dark lines in the external or distal part of the coxa and sternum.The number of trichobothria on the pedipalp manus ventral surface is 4 (3 *V + Et* 1); the number of trichobothria on the pedipalp patella ventral surface is 8(in 87.29% of examined pedipalps); the number of trichobothria on pedipalp patella external surface is: *eb* = 4, *eba* = 4, *esb* = 2, *em* = 4, *est* = 4, *et* = 6 ( in 77.96% of examined pedipalps).The pectinal teeth count is 9 (in 80.55% of examined pectines) in males, 7 (in 68.29% of examined pectines) in females.The telson vesicle in males is considerably more swollen than in females: average L/H ratio of the vesicle is 2.07 in male and 2.30 in females.Chela with a notch on fixed finger and scalloping of the movable finger in adult males, obsolete in females.Dorsal patellar spur well developed. Average L/W ratio of the chela is 2.35 in males and 2.48 in females. Average length/posterior width ratio of the carapace is 0.98. All carinae on pedipalps are strongly distinct and dark, in contrast with clear color of tegument.Average value of the length from center median eyes to anterior margin of the carapace is 42.47% of the carapace length. Average value of the length from center median eyes to posterior margin of the carapace is 57.53% of the carapace length.

##### Description of the holotype male.

***Coloration*:** Very light brownish with carapace and pedipalps little darker, legs, telson and chelicerae are lighter. The carinae are dark, distinctly brownish-blackish, especially on pedipalps. Dark lines in the external or distal part of the coxa and sternum. Granulometry on the femora of the legs, especially ventrally, dark. The sternites, pectines and genital operculum are very light brownish-white.

***Carapace*:** Length 4.11 mm; posterior width 4.14. Very finely granulated. Distance from the center of the median eyes to the anterior margin of the carapace is equivalent to 42.33% of the prosoma; the length from the center of the median eyes to the posterior margin of the carapace is equivalent to 57.67% of the prosoma.

***Mesosoma*:**Tergites veryfinely granulated; sternites finely punctate. The area of overlap between the sternites is lighter in color. Pectinal teeth count is 9-9. The spiracles are very small, oval-shaped and it is inclined about 45° downwards towards outside.

***Metasoma*:** Medium size with respect to body length. Dorsal carinae from segment I-IV are granulated, exhibiting dark granules, obsolete on the segment V; ventromedian carinae from segment I-IV absent; ventromedian carinae on segment V are formed by fine granules; ventrolateral carinae on segment I absent, on segments II and III smooth, on segment IV is formed by small dark granules, on segment V is formed by dark granules; all intercarinal spaces are finely granulated.

***Telson*:** Vesicle weakly swollen; smooth, with ventral setae of different sizes; telson height 1.38; telson length 3.75; vesicle length 2.85; vesicle width 1.38; L/H ratio of the vesicle 2.06.

***Pectines*:**Pectinalteeth count 9-9; middle lamellae count 6-6.

***Genital operculum*:**Partially divided with genital papillae protruding; a few microsetae present.

***Sternum*:**Pentagonal shape, type 2. Length similar to width, deep posterior emargination.

**Figure 1.**
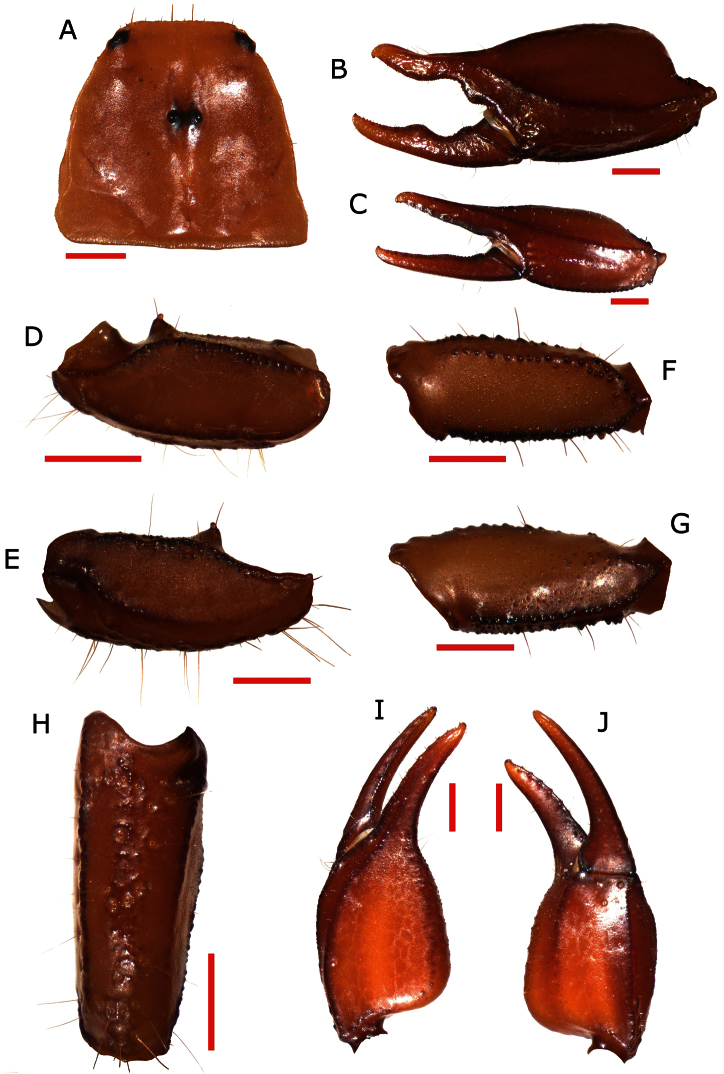
**A** carapace **B** external view of chela of the adult male **C** external view of chela of the adult female **D** ventral view of pedipalp patella **E** dorsal view of pedipalp patella **F** ventral view of pedipalp femur **G** dorsal view of pedipalp femur **H** view of external surface of pedipalp patella **I** dorsal view of chela **J** ventral view of chela.

***Pedipalp*:** Coxa and trochanter with strong granulation.Femur: dorsal internal carinae tuberculate;dorsal external carinae formed by tubercles, slightly serrulated; intercarinal spaces granulated; external median carinae serrulate, anterior median formed by hardly conical tubercle. Patella length 3.54; patella width 1.38; dorsal internal carinae crenulate to tuberculate; dorsal external carinae low, from rough to crenulate; Ventral external carinae crenulate; ventral internal carinae from serrulate to tuberculate; dorsal intercarinal tegument with granules of increased size from proximal to distal area; ventral intercarinal tegument from smooth to minutely granulate with a few bigger granules near to ventral internal carinae; internal intercarinal tegument uniformly finely granulate. Dorsal patellar spur averagely developed (Fig. 1E). Chelal carina *D_1_* isdistinctly strong, dark and from smooth to rough; *D_4_* is rough with a few low granules in proximal area;*V****_1_*** isdistinctly strong, from rough to crenulate and dark; *V_3_* dark on ¾ of length, mostly smooth with a few scattered minuscule granules; external carina rough and dark; intercarinal tegument from smooth to rough except between carinae *D_4_* and *V_3_*, granulate. Movable finger dentition: MD form a straight line of very small denticles closely spaced with a DD on the distal tip; OD formed of 7 denticles on movable finger and 6 denticles on fixed finger, immediately outside of MD, their size increases progressively but the terminal denticle is not very pronounced; ID formed of 7 denticles on movable finger and 6 denticles on fixed finger, spaced from MD, their size increases progressively but the terminal denticle is not very pronounced; IAD on both movable and fixed finger formed of 4 small denticles; L/W ratio of the chela 2.35

***Trichobothria*:** Chela trichobothria series *V* standard: *V* = 4-4 (3 *V+ Et*1); patella ventral (*P*v): 8-8; Patella external (*P*e): *et* = 6-6, *est* = 4-4, *em* = 4-4, *esb* = 2-2, *eba* = 4-4, *eb* = 4*-*4.

***Legs*:** legs with two pedal spurs. Tarsal ventral row with 10-12 stout spinules (including the ventral distal spinule pair); 3 flanking pairs of tarsal setae adjacent to the ventral spinules row.Basitarsus with 6-7 prolateral stout spinules on leg pair I; 4-3 prolateral stout spinules on leg pair II; absent on leg pair III and IV. Dark granulation present above leg femora, mostly ventrally; on the dorsal leg femora I it is weakly marked and of lighter color.

***Chelicerae***: smooth, without marbling, with darker teeth; the dorsal distal tooth is smaller than the ventral distal tooth; ventral edge is smooth with brush-like setae on the inner part; dorsal edge has five teeth: one distal, two small subdistal, one big median and a small basal; fixed finger has four teeth: one distal, one subdistal, one median and one basal; the median and the basal are in a fork arrangement; the internal edge has brush-like setae.

***Variation*:** The variation observed in 59 studied specimens (18 males, 41 females) as follows: pectinal teeth in males: 8-8 (2/18), 8-9 (1/18), 9-9 (13/18), 9-10 (2/18); females: 6-6 (1/41), 6-7 (3/41), 7-7 (23/41), 8-7 (7/41), 8-8 (6/41), 8-9 (1/41); pedipalp patella trichobothria *Pv*: 7-7 (2/59), 8-7 (10/59), 8-8 (46/59), 9-8 (1/59); pedipalp patella trichobothria *Pe*: *et* = 5-5(6/59), 5-6 (14/59), 6-6 (39/59); *est* = 3-4 (2/59), 4-4 (57/59), *em* = 4-4 (59/59), *esb*= 2-2 (59/59), *eba* = 4-4 (59/59), eb = 4-4 (59/59). The variation in the trichobothrial pattern is within the standard values of variability and shows the stability of diagnostic characters.

***Hemispermatophore*:** Well developed lamina with well visible basal constriction, tapered distally; truncal flexure present and well developed; capsular lobe complex well developed, with acuminate process; ental channel spinose distally, exhibiting 8-12 delicate spines.

**Figure 2.**
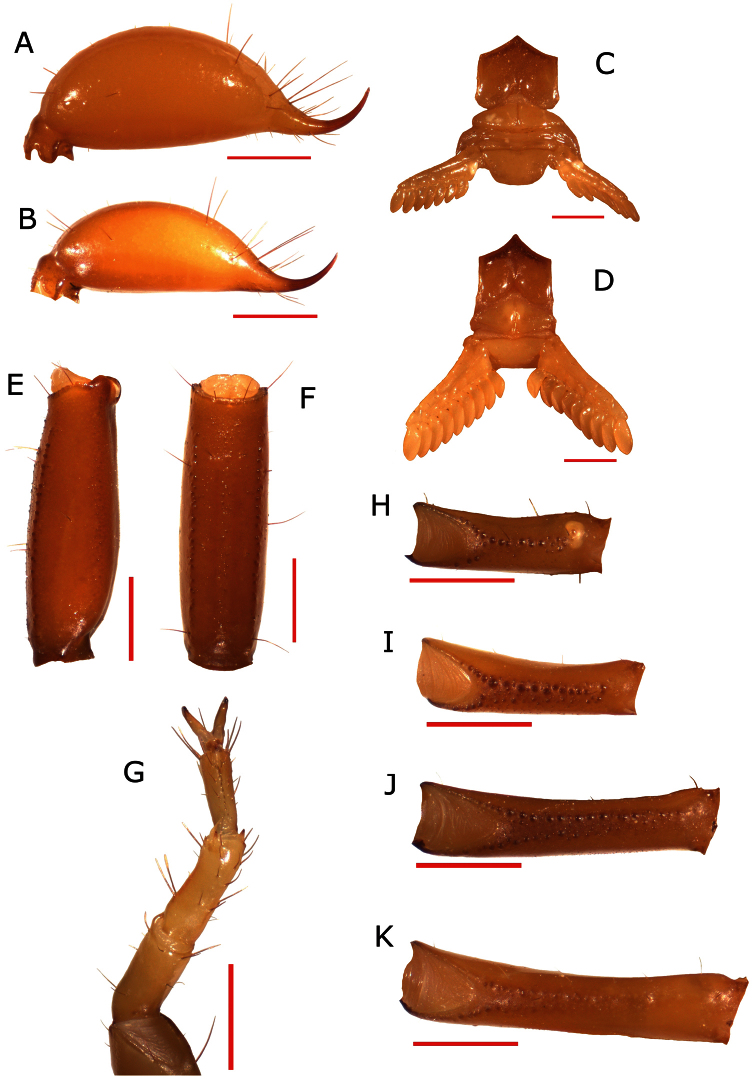
**A** telson of adult male **B** telson of adult female **C** sternopectinal area of adult female **D **sternopectinal area of adult male **E** latero-dorsal view of the metasomal segment V **F** ventral view of the metasomal segment V **G** tarsus and basitarsus **H** leg femur I **I** leg femur II **J** leg femur III **K** leg femur IV.

**Figure 3.**
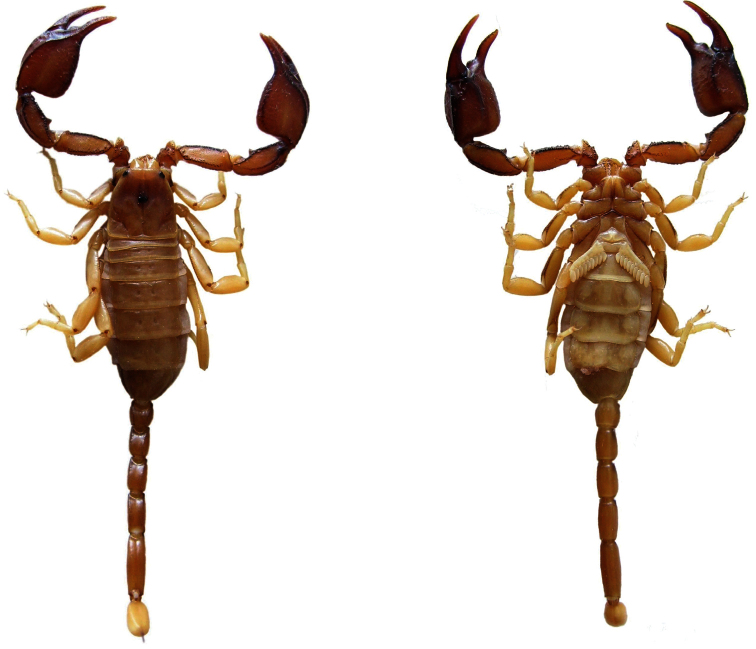
Dorsal and ventral views of *Euscorpius rahsenae* sp. n. male.

**Figure 4.**
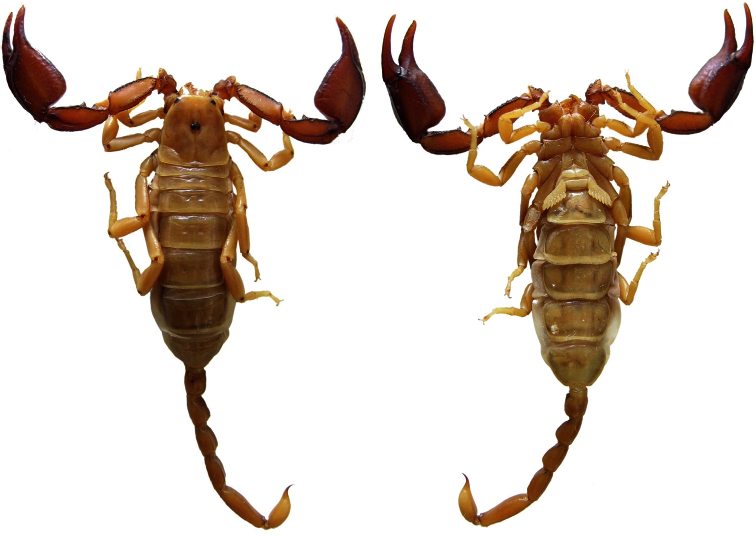
Dorsal and ventral views of *Euscorpius rahsenae* sp. n. female.

**Figure 5.**
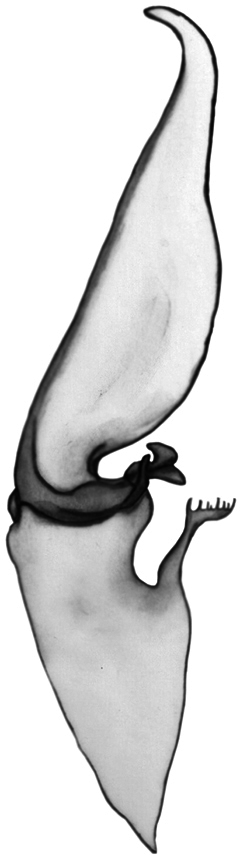
Left hemispermatophore of *Euscorpius rahsenae* sp. n.

**Figure 6.**
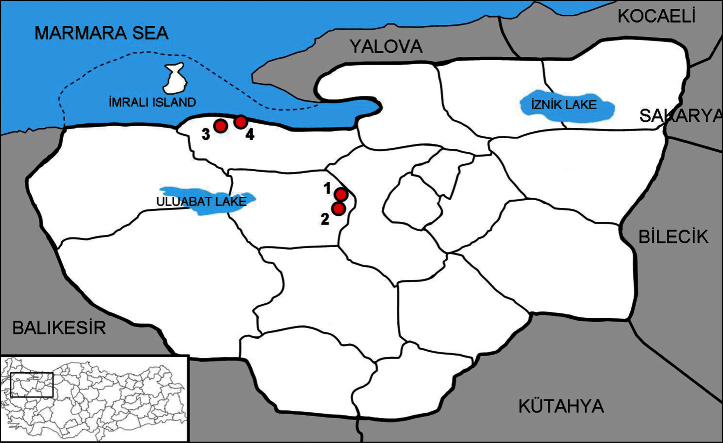
Sampling map of *Euscorpius rahsenae* sp. n. in Bursa Province

**Figure 7.**
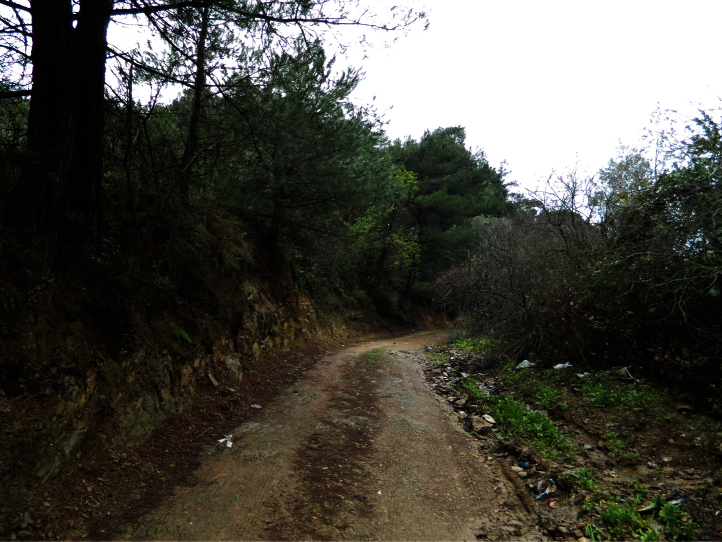
The pine forest habitat in Tirilye Village.

**Figure 8.**
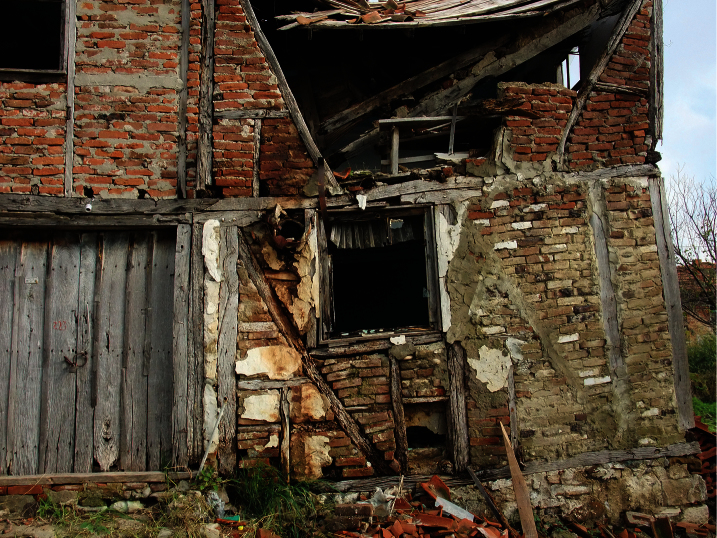
The ruined building habitat in Yalıçiftlik Village.

**Table 1.** Measurements (in mm) of male holotype and female paratype of *Euscorpius rahsenae* sp. n.

		**Holotype**	**Paratype female**
**Total**	Length	28.86	28.21
**Carapace**	Length	4.11	4.06
	Posterior width	4.14	4.14
**Metasoma**	Length	11.40	10.34
**Segment I**	Length	1.50	1.32
	Width	1.49	1.38
**Segment II**	Length	1.80	1.62
	Width	1.26	1.13
**Segment III**	Length	1.98	1.85
	Width	1.20	1.08
**Segment IV**	Length	2.34	2.16
	Width	1.14	1.02
**Segment V**	Length	3.78	3.39
	Width	1.14	1.02
**Telson**	Length	3.75	3.30
**Vesicle**	Length	2.85	2.31
	Width	1.38	1.06
	Height	1.38	1.02
**Aculeus**	Length	0.90	0.99
**Femur**	Length	3.42	3.42
	Width	1.32	1.32
**Patella**	Length	3.54	3.53
	Width	1.38	1.44
**Chela**	Length	7.20	6.96
	Width	3.06	2.72
**Movable finger**	Length	4.20	3.90

## Discussion and comparison

Karataş (2006) reported two assemblages of populations of the subgenus *Euscorpius* from Turkey as “*Euscorpius* sp.1” and “*Euscorpius* sp.2”. The first has been reported from Bursa, Çanakkale, İstanbul, İzmir, and Sinop Provinces; the second has been reported from Antalya and Muğla Provinces. *Euscorpius rahsenae* sp. n. occurs within the area of the first assemblages (Marmara Region), however, in this work, we describe as *Euscorpius rahsenae* sp. n. only the population of particularly light specimens with a strong contrast of dark carinae, that occurs in the Bursa Province. We are conducting further studies to understand the relationship between *Euscorpius rahsenae* sp. n. and the reddish populations found further north.

Karataş (2006) compared “*Euscorpius* sp.1” with *Euscorpius koschewnikowi* from Greece, observing some differences; for this reason we also compared *Euscorpius rahsenae* sp. n. with *Euscorpius koschewnikowi*. Note that Karataş (2006), among the differences between “*Euscorpius* sp.1” and *Euscorpius koschewnikowi*, reported that in specimens studied by her,* V4* was situated on the *ventral* surface, internally from the exteroventral carina while according to Fet and Soleglad (2002) the trichobothrium *V4* is situated on the external surface, removed from the exteroventral carina in *Euscorpius koschewnikowi*. *Euscorpius rahsenae* sp. n. specimens, as well as those from İstanbul that coincide with “*Euscorpius* sp.1” of Karataş (2006), have the trichobothrium *V4* situated on the external surface, as is normal in the subgenus *Euscorpius*. It is possible that Karataş (2006) has misinterpreted the trichobothrial nomenclature of the chela.

*Euscorpius koschewnikowi* was described by Birula (1900) from Mt Athos, Agion Oros, in the northeast of Greece. Kinzelbach (1975) synonymized it with *Euscorpius carpathicus* but Fet and Soleglad (2002) redescribed this form, elevating it to species status. *Euscorpius koschewnikowi* is a medium to large sized species (up to 46 mm), medium to dark brown in color, slender appearance with well developed dorsal patellar spur and all metasoma segments longer than wide. In addition, according to Fet and Soleglad (2002) the exceptionally slender and smooth metasoma are key diagnostic characters of this species. *Euscorpius rahsenae* sp. n. differs by *Euscorpius koschewnikowi* for the colour very lighter, brownish-ivory, smaller average size, the metasomal segments not particularly smooth and the first segment not always longer than wide, especially in females (average L/W ratio 1.03 in males, 0.98 in females).

The only valid species belonging to the subgenus *Euscorpius* in Turkey is *Euscorpius avcii*. This species was recently described from Dilek Peninsula as an oligotrichous, small *Euscorpius*, with a length of 24-28 mm, light brown to brown-reddish colored with the carapace and pedipalps darker and legs and telson lighter (Tropea et al. 2012). It is possible to differentiate this species from *Euscorpius rahsenae* sp. n. as follows: the color of *Euscorpius avcii* is reddish brown while *Euscorpius rahsenae* sp. n. is very light brown-ivory with a strong contrast dark color of the carinae; *Euscorpius avcii* is on average smaller than *Euscorpius rahsenae* sp. n. (24–28 cm and 27–34 mm respectively); the pectinal teeth count in *Euscorpius avcii* is 7 in females and 8 in males while in *Euscorpius rahsenae* sp. n. is usually 7 in females and 9 in males; *Pv* count is usually 7 in *Euscorpius avcii* and 8 in *Euscorpius rahsenae* sp. n.; *Pe-et* series is generally 5 in *Euscorpius avcii* and 6 in *Euscorpius rahsenae* sp. n.; hemispermatophore exhibiting 6 delicate spines in *Euscorpius avcii*, 8–12 in *Euscorpius rahsenae* sp. n.

The other forms of the subgenus *Euscorpius* are obviously different species and geographically distant. Below, we compare *Euscorpius rahsenae* sp. n. to some other forms present in the Aegean area: *Euscorpius sicanus* (C. L. Koch, 1837), *Euscorpius carpathicus candiota* Birula, 1903, *Euscorpius carpathicus scaber* Birula, 1900, *Euscorpius carpathicus ossae* Di Caporiacco, 1950 and *Euscorpius carpathicus aegaeus* Di Caporiacco, 1950.

*Euscorpius sicanus* complex is widespread in mainland Greece and some Aegean islands (Fet et al. 2003), and can be easily distinguished from *Euscorpius rahsenae* sp. n. by the trichobothrial series *eb* = 5 in *Euscorpius sicanus* complex and *eb* = 4 in *Euscorpius rahsenae* sp. n.

*Euscorpius carpathicus candiota* is a light colored species, described from Crete. It can be distinguished by the *Euscorpius rahsenae* sp. n. by the higher number of trichobothria and pectinal teeth; *Pv* = 9/10 (usually 10), *Pe-et* = 6/8 (generally 7) and pectinal teeth count 9 to 10 in males (generally 10) and 7 to 8 in females, compared to *Pv* = 8, *Pe-et* = 6 and pectinal teeth count 9 in males and 7 in females.

*Euscorpius carpathicus scaber* is a scorpion from the northern Aegean area, with a dark coloration, an higher number of pectinal teeth, an higher trichobothrial pattern, and in addition, a body totally covered by granules of various size, as also the name suggests, whereas *Euscorpius rahsenae* sp. n. is light yellowish-brown, without a particularly accentuated granulation. *Euscorpius carpathicus ossae* is an oligotrichous form, dark brown in colour with lighter legs and telson. It was described from Mount Ossa, in Thessaly. This form can be distinguished mainly by the dark colour, the *Pv*=7 and *et*=5, compared with *Pv*=8 and Pe-*et*=6 of *Euscorpius rahsenae* sp. n. *Euscorpius carpathicus aegaeus* is a light colored form described from the island of Antiparos, in the central-southern part of the Aegean Sea. Probably it is endemic in few islands in the central-south Aegean Sea. In addition, it is described as uniformly light yellow colored and females with pectinal teeth count 8 (Di Caporiacco 1950), while *Euscorpius rahsenae* sp. n. has carapace and pedipalpslittle darker, legs, telson and chelicerae lighter with carinae dark, distinctly brownish-blackish and pectinal teeth count 7 in females.

**Table 2.** Trichobothrial counts of *Euscorpius* species discussed in this paper.

**Species**	***Pv***	***Pe - et***	***Pe - est***	***Pe - em***	***Pe - esb***	***Pe - eba***	***Pe - eb***
*Euscorpius rahsenae* sp.n.	8	5-6 (6)	4	4	2	4	4
*Euscorpius avcii*	7	5-6 (5)	4	4	2	4	4
*Euscorpius koschewnikowi*	8	5-6	4	4	2	4	4
*Euscorpius carpathicus aegaeus*	7-8 (8)	5-6 (6)	4	4	2	4	4
*Euscorpius carpathicus ossae*	7	5	4	4	2	4	4
*Euscorpius carpathicus scaber*	7-10(8/9)	6	4	4	2	4	4
*Euscorpius carpathicus candiota*	9-10(10)	6-7(7)	4	4	2	4	4

## Ecology

Some specimens of *Euscorpius rahsenae* sp. n. were collected from city center (Beşevler and Çiftehavuzlar) and in a ruined building in Yalıçiftlik Village (Mudanya District) of Bursa Province. It shows that *Euscorpius rahsenae* sp. n. penetrates to human settlements and is an anthropotelerant species.

A large part of Mudanya is an urban area, but the Tirilye locality (Mudanya) has vegetation composed of red pine (*Pinus brutia* Ten.), torch pine (*Pinus nigra* Arn. subsp. *pallasiana* (Lamb)), olive trees (*Olea europea* L.), and maquis vegetation(*Quercus* sp., *Erica arborea* L., *Juniperus oxycedrus* L., *Phillyrea latifolia* L., *Pistacia lentiscus* L., *Cistus* spp., as main shrubs). The specimens were collected in this locality during night trips with UV light when sitting in cracks of the earthen wall along the roadsides in the forest.

## Supplementary Material

XML Treatment for Euscorpius rahsenae

## References

[B1] Di Caporiacco L (1950) Le specie e sottospecie del genere “*Euscorpius*” viventi in Italia ed in alcune zone confinanti, Memorie/Atti della Accademia Nazionale dei Lincei, serie VIII, volume II, sezione III, fascicolo 4: 159–230.

[B2] Fet V Soleglad ME (2002) Morphology analysis supports presence of more than one species in the “*Euscorpius carpathicus*” complex (Scorpiones: Euscorpiidae). Euscorpius 3: 1-51.

[B3] Fet V Soleglad ME Gantenbein B Vignoli V Salomone N Fet EV Schembri PJ (2003) New molecular and morphological data on the *Euscorpius carpathicus* species complex (Scorpiones: Euscorpiidae) from Italy, Malta, and Greece justify the elevation of *Euscorpius carpathicus sicanus* (C.L. Koch, 1837) to the species level. Revue suisse de Zoologie 110: 355-379.

[B4] Gantenbein B Fet V Largiadèr, CR Scholl A (1999) First DNA phylogeny of *Euscorpius* Thorell, 1876 (Scorpiones: Euscorpiidae) and its bearing on taxonomy and biogeography of this genus. Biogeographica (Paris) 75 (2): 49-65.

[B5] Hadži J (1930) Die europäischen Skorpione des Polnischen Zoologischen Staatsmuseums in Warszawa. Annales Musei Zoologici Polonici 9 (4): 29-38.

[B6] Hjelle JT (1990) Anatomy and morphology. In: Polis GA (Ed.). Biology of Scorpions. Stanford University Press, Stanford, CA: 9-63.

[B7] Karataş A (2006) Distribution of the “*Euscorpius carpathicus*” complex (Scorpiones: Euscorpiidae) in Turkey. Serket 10 (1): 1-8.

[B8] Kinzelbach R (1975) Die Skorpione der Ägäis. Beiträge zur Systematik, Phylogenie und Biogeographie. Zoologische Jahrbücher, Abteilung für Systematik 102: 12-50.

[B9] Kinzelbach R (1982) Die Skorpionssammlung des Naturhistorischen Museums der Stadt Mainz. Teil I: Europa und Anatolien. Mainzer Naturw. Archiv 20: 49-66.

[B10] Koç H Yağmur EA (2007) Dilek Yarımadası Milli Parkı (Söke-Kuşadası, Aydın) akrep faunası. Ekoloji Dergisi 65: 52-59.

[B11] Koch CL (1837) Die Arachniden. C. H. Zeh‘sche Buchhandlung, Nürnberg 3 (6): 105-115.

[B12] Lacroix J-B (1995) *Euscorpius* (*E*.) *mingrelicus* Kessler, 1876 en Turquie anatolienne (Arachnida: Scorpionida). Arachnides 26: 4-6.

[B13] Laurie M (1896) Further notes on the anatomy of some scorpions, and its bearing on the classification of the order. Ann. Mag. nat. Hist. (6) 18: 121–133. doi: 10.1080/00222939608680422

[B14] Schenkel E (1947) Einige Mitteilungen über Spinnentiere. Revue suisse de Zoologie 54 (1): 13–16.

[B15] Sissom WD (1990) Systematics, biogeography and paleontology. In: Polis GA (Ed.) The Biology of Scorpions, Stanford University Press, 64–160.

[B16] Soleglad ME Fet V (2003) The scorpion sternum: structure and phylogeny (Scorpiones: Orthosterni). Euscorpius 5: 1-33.

[B17] Soleglad ME Sissom WD (2001) Phylogeny of the family Euscorpiidae Laurie, 1896: a major revision. In: Fet V Selden PA (Eds). Scorpions 2001, In Memoriam Gary A. Polis, British Arachnological Society, Burnham Beeches, Bucks, UK: 25-112.

[B18] Stahnke HL (1970) Scorpion nomenclature and mensuration. Entomol. News 81: 297-316. 5417256

[B19] Thorell T (1876) On the classification of scorpions. Annals and Magazine of Natural History 4 (17): 1-15. doi: 10.1080/00222937608681889

[B20] Tolunay A (1959) Zur Verbreitung der Skorpione in der Türkei. Zeitschrift für angewandte Entomologie 43: 366-370.

[B21] Tropea G Yağmur EA Koç H Yeşilyurt F Rossi A (2012) A new species of *Euscorpius* Thorell, 1876 (Scorpiones, Euscorpiidae) from Turkey. ZooKeys 219: 63-80. doi: 10.3897/zookeys.219.3597 PMC3433697 22977350

[B22] Tropea G Rossi A (2011–2012) A new species of *Euscorpius* Thorell, 1876 from Corfu, with notes on the subgenus *Euscorpius* in Greece (Scorpiones: Euscorpiidae). Onychium 9: 27–37

[B23] Vachon M (1951) A propos de quelques scorpions de Turquie collectés par M. le Professeur Dr. Curt Kosswig. İstanbul Üniversitesi Fen Fakültesi Mecmuası 16: 341-344.

[B24] Vachon M (1974) Étude des caractères utilisés pour classer les familles et les genres de Scorpions (Arachnides). 1. La trichobothriotaxie en Arachnologie, Sigles trichobothriaux et types de trichobothriotaxie chez les Scorpions. Bulletin du Museum D'Histoire Naturelle, Paris 140: 857-958.

